# Seizure Diaries and Forecasting With Wearables: Epilepsy Monitoring Outside the Clinic

**DOI:** 10.3389/fneur.2021.690404

**Published:** 2021-07-13

**Authors:** Benjamin H. Brinkmann, Philippa J. Karoly, Ewan S. Nurse, Sonya B. Dumanis, Mona Nasseri, Pedro F. Viana, Andreas Schulze-Bonhage, Dean R. Freestone, Greg Worrell, Mark P. Richardson, Mark J. Cook

**Affiliations:** ^1^Department of Neurology, Mayo Foundation, Rochester, MN, United States; ^2^Department of Medicine, Graeme Clark Institute and St Vincent's Hospital, The University of Melbourne, Fitzroy, VIC, Australia; ^3^Seer Medical, Melbourne, VIC, Australia; ^4^Epilepsy Foundation, Landover, MD, United States; ^5^School of Engineering, University of North Florida, Jacksonville, FL, United States; ^6^Institute of Psychiatry, Psychology and Neuroscience, King's College London, London, United Kingdom; ^7^Faculty of Medicine, University of Lisbon, Lisboa, Portugal; ^8^Faculty of Medicine, Epilepsy Center, Medical Center, University of Freiburg, Freiburg, Germany

**Keywords:** wearable devices, seizure detection, seizure forecasting, multidian cycles, machine learning, epilepsy

## Abstract

It is a major challenge in clinical epilepsy to diagnose and treat a disease characterized by infrequent seizures based on patient or caregiver reports and limited duration clinical testing. The poor reliability of self-reported seizure diaries for many people with epilepsy is well-established, but these records remain necessary in clinical care and therapeutic studies. A number of wearable devices have emerged, which may be capable of detecting seizures, recording seizure data, and alerting caregivers. Developments in non-invasive wearable sensors to measure accelerometry, photoplethysmography (PPG), electrodermal activity (EDA), electromyography (EMG), and other signals outside of the traditional clinical environment may be able to identify seizure-related changes. Non-invasive scalp electroencephalography (EEG) and minimally invasive subscalp EEG may allow direct measurement of seizure activity. However, significant network and computational infrastructure is needed for continuous, secure transmission of data. The large volume of data acquired by these devices necessitates computer-assisted review and detection to reduce the burden on human reviewers. Furthermore, user acceptability of such devices must be a paramount consideration to ensure adherence with long-term device use. Such devices can identify tonic–clonic seizures, but identification of other seizure semiologies with non-EEG wearables is an ongoing challenge. Identification of electrographic seizures with subscalp EEG systems has recently been demonstrated over long (>6 month) durations, and this shows promise for accurate, objective seizure records. While the ability to detect and forecast seizures from ambulatory intracranial EEG is established, invasive devices may not be acceptable for many individuals with epilepsy. Recent studies show promising results for probabilistic forecasts of seizure risk from long-term wearable devices and electronic diaries of self-reported seizures. There may also be predictive value in individuals' symptoms, mood, and cognitive performance. However, seizure forecasting requires perpetual use of a device for monitoring, increasing the importance of the system's acceptability to users. Furthermore, long-term studies with concurrent EEG confirmation are lacking currently. This review describes the current evidence and challenges in the use of minimally and non-invasive devices for long-term epilepsy monitoring, the essential components in remote monitoring systems, and explores the feasibility to detect and forecast impending seizures *via* long-term use of these systems.

## Introduction

It has long been recognized that seizures occur more frequently than self-reported, though the scale of this underestimation has only recently been appreciated (1–4). This has complicated our ability to provide optimal care and safety strategies (5, 6), and casts uncertainty on the validity of therapeutic strategies and clinical trials results (4).

Wearable sensing devices are increasing in popularity both in the general community and through medical applications such as seizure detection. However, there is insufficient data relating to the clinical utility and reliability of these systems (7). There are also significant concerns around data security, privacy, and data ownership (8), and questions relating to the optimal software, hardware, and data transmission systems. Additionally, there are several separate issues to consider with wearable devices: how the data is acquired, what systems can be used to achieve this acquisition, and how the data may be used to provide more sophisticated feedback to individuals and their caregivers. Wearable devices may also facilitate reliable forecasts of seizure likelihood, providing the potential for people with epilepsy to take fast-acting medications or modify activities in anticipation of an impending seizure (9–11).

Chronically implanted intracranial electroencephalography (EEG) systems have resulted in dramatic insights into the dynamics and underlying rhythms of epileptic activity and seizures (2, 12–19) but are not suitable for widespread use because of issues relating to cost and risk, and are limited in spatial sampling. In addition to unreported seizures, these devices also detect a large number of electrographic seizure patterns without clear behavioral correlates. However, this electrographic epileptic activity is highly relevant to epilepsy management and seizure forecasting, and chronic EEG remains vital to develop and validate standalone wearable systems. These recent studies suggest that the aims of seizure forecasting might be achieved through capturing data, which represent trends and associations in individuals and populations, harnessing the strength of multiple sources, and applying recently developed strategies in machine-learning to combine this information and generate measures of seizure risk. Seizure forecasting using these techniques might ultimately become a useful way for individuals to manage daily activities, and for clinicians to accurately judge the efficacy of therapies.

## Seizure Reporting and Detection

An obstacle currently to clinical management of epilepsy is the scarcity of accurate, reliable information available to the physician when diagnosing a seizure disorder and identifying therapeutic options. Because seizure events are infrequent, the physician may not be able to directly observe events, and must rely on the individual, caregivers, and other witnesses to describe events, identify potential precipitants, report their frequency, and discuss the impact on the person's daily life (20). In-hospital diagnostic tests are expensive and may produce a diagnosis of epilepsy, psychogenic non-epileptic events, or syncope, or may be diagnostically inconclusive. Even when a clear diagnosis of epilepsy is established, the limited availability of accurate information hinders effective therapy (1). People with epilepsy may be partly or fully amnestic to seizures (21, 22), individuals may be unable to provide an accurate account of seizure occurrence and severity (23–25), and witness accounts of epileptic and behavioral events are often unreliable (26–28). Changes in seizure frequency and severity, sometimes due to poor medication adherence (29), may increase a person's risk of SUDEP (30), status epilepticus (31), or injury during daily activities. Currently, physicians and caregivers have no way to identify increases in seizure frequency and/or severity between office visits. Many studies confirm that people under-report their seizures for a variety of reasons [summarized by Elger and Hoppe (3)]. A study of an implanted EEG monitoring device for seizure forecasting (2), compared to monthly self-reported seizure diaries to ambulatory intracranial EEG, found vast discrepancies in seizure reports, with some subjects reporting no seizures in months where the device recorded hundreds of clinical and electrographic seizures. Compared to long-term ambulatory EEG monitoring, individuals were found to report less than half of measured seizures (3), and people with epilepsy enrolled in clinical medication trials were aware of their own seizure underreporting in post-study telephone interviews (4).

Objective data characterizing seizure counts (32) and severity (33) could be obtained using devices capable of capturing and storing EEG or other biosignals that indicate seizure occurrence. Invasive, implanted EEG (33) devices with limited capabilities are available currently. The NeuroPace RNS device (16, 34) is clinically available and provides responsive neurostimulation to suppress seizures in focal epilepsy, but also has the ability to record and store limited data segments on the device. Investigational devices like the Medtronic PC+S have a similar capabilities (35, 36) but are used only in limited research applications. The limited data capacity of such devices makes it difficult to evaluate detection sensitivity because there is no way to confirm that *all* seizures have been identified, although identified and stored events can be confirmed as electrographic seizures (specificity). Finally, minimally invasive subscalp EEG devices are emerging as potential alternatives for continuous EEG recording, providing a balance between signal quality and user acceptability. One subscalp system (24/7 EEG^TM^ SubQ) has been CE-marked for epilepsy monitoring and diagnosis, following a cumulative 490 day trial (nine patients, up to 90 days each) demonstrating its safety and feasibility (37). Automatically assisted, visual identification of electrographic seizures has also been demonstrated in the ultra long-term setting (>6 months) with this system, with excellent sensitivity but low specificity (38).

### Non-invasive Seizure Monitoring

**Figure 1:**
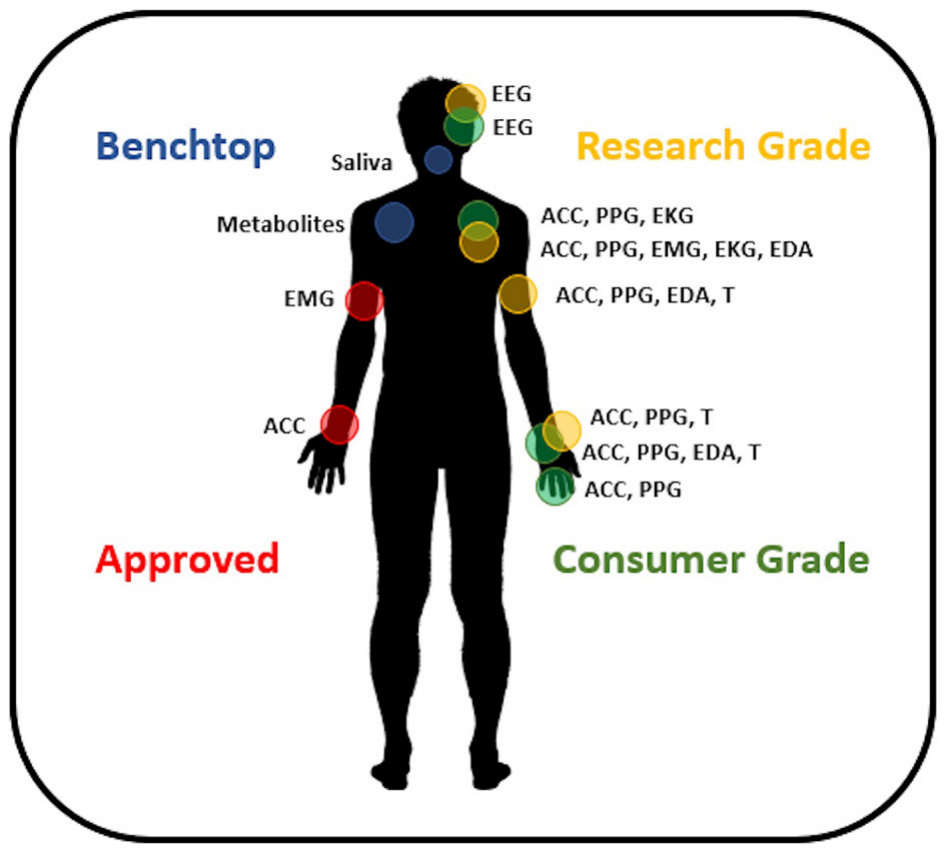
Available wearable devices for seizure management. Approved devices include sensor systems CE Marked and/or FDA approved for epilepsy. Research grade devices are commercially available and provide accurate, high-quality data. Consumer grade devices are commercially available sensors designed around applications where data accuracy is not crucial and may utilize interpolation or estimation methods to provide information to the user. Benchtop devices are innovative sensors under development and not available commercially. EEG, electroencephalography; ACC, accelerometry; PPG, photoplethysmography; EKG, electrocardiography; EMG, electromyography; EDA, electrodermal activity; T, temperature.

Non-invasive, wearable biosensors have the greatest immediate potential to meet the needs of the majority of people with epilepsy. The availability of inexpensive miniaturized electronic components, wireless data telemetry, and rechargeable battery technology has given rise to a large number of lightweight, wearable sensors. Currently, sensors are commercially available to measure continuous, non-invasive photoplethysmography (PPG, to measure the blood volume pulse signal), electromyography (EMG), accelerometry, EEG, electrocardiography (EKG), electrodermal activity (EDA), and skin temperature in a range of form factors. A summary of available sensors is given in Figure 1 (see also Panels in Appendix). Most individuals are familiar with the accelerometers and optical PPG sensors included in consumer electronics like smart watches and fitness monitors. These inexpensive sensors with sophisticated data processing algorithms on cloud-based data management systems are capable of tracking sleep and exercise rates based on accelerometry (39–41), although the accuracy of sleep staging with these devices is unclear. Variable accuracy has been found as well in tracking heart rates from wrist-worn PPG sensors (42). Nevertheless, wearable biosensors remain of interest for epilepsy management, as changes in sleep quality (43), exercise (i.e., heart rate and motion tracking) (44), and stress (i.e., heart rate variability, EDA) (45, 46) may all trigger seizure onset for some people.

Currently, there are two wearable sensors approved by the FDA and EU for detecting convulsive seizures: the first, a wrist-worn smartwatch (Empatica Embrace, Boston MA), uses accelerometry and EDA to detect the subject's movements and maintains a Bluetooth link to the subject's smartphone, where an application telemeters data and detections to cloud servers and issues caregiver alerts for seizures (47). The Empatica Embrace was CE Marked in 2016 and FDA approved in 2018. The second device is attached by an adhesive patch affixed to the subject's bicep and identifies changes in EMG to detect convulsions (BrainSentinel SPEAC, San Antonio TX). This device also has a cloud-based data platform and can send caregiver alerts and was CE Marked in 2013 and FDA approved in 2017 (48). Other CE-Marked devices are available (Biovotion Everion, ByteFlies Sensor Dots, Livassured NightWatch, Epi-Care Free), and studies of performance at detecting seizures are ongoing. Detection of convulsive or motor seizures is relatively easier than other seizure types (49), and studies are beginning to address these other more difficult semiologies, but only with modest success to date (50–52). As detection performance improves, approved devices may become an adjunct measure of seizure activity for anti-seizure medication trials. These would likely initially be used exclusively for the detection of tonic–clonic seizures, as wearable sensors are most performant for this seizure type.

Most commercially available sensors do not have regulatory approval for use in epilepsy, and these sensors span an array of form factors and capabilities. The majority of commercial smartwatches now carry accelerometric and PPG sensors, which could be useful in tracking seizures (53), and smartwatch and smartphone applications have been developed for this purpose. Rigorous testing data is needed, however, and until clear estimates of sensitivity and specificity under a range of conditions are established, wearable systems should not be considered reliable sources of clinically actionable information (54). Apple and FitBit's consumer grade wearables have FDA and CE approval for cardiovascular monitoring currently, and as data accumulates, regulatory approvals for epilepsy could be possible. Devices aimed at the clinical research market are available in a wrist-watch form factor (Empatica E4, Geneactiv), and this form factor is often rated well by people with epilepsy for comfort and ease of use (55). Smart devices in a ring form factor (e.g., Oura and Motiv) can collect accelerometry and finger PPG. The small size of these devices severely limits their battery capacity, and most devices do not incorporate real-time Bluetooth data linkage. Ring devices may be useful for seizure diary applications, or to provide estimates of sleep quality and other factors to forecasting algorithms, but currently are not able to provide physiological data in real time. PPG data quality is adversely affected by subject movement, and wrist and hand-worn PPG sensors may suffer due to limb movements. Recently published results of commercially available wearable sensors in seizure detection are summarized in Table 1.

**Table 1:** Sensitivity and false alarm rates for detection of seizures with wearable biosensors.

**Study**	**Device**	**Signal(s)**	**Environment**	**Seizure type**	**Patients (seizures)**	**Sensitivity (%)**	**False alarms per day**
Beniczky (56)	IctalCare EDDI	EMG	EMU	GTCS	71 (32)	93.8	0.67
Halford (57)	BrainSentinel SPEAC	EMG	EMU	GTCS	199 (46)	76	2.52
					149 (29)^a^	100^a^	1.44^a^
Onorati (49)	Empatica E4	ACC,EDA	EMU	GTCS	69 (22)	94.5^b^	0.2^b^
Vandencasteele (58)	180° eMotion Faros	EKG	EMU	CP (FT)	11 (47)	70	51.6
	Empatica E4	PPG				32	43.2
Johansson (59)	Shimmer3, custom device	ACC	EMU	TCS	8 (10)^c^	100^b^	1.2^b^
Heidberg (51)	Empatica E3	ACC, EDA	EMU	Multiple	8 (55)	89.1^d^	18.1^e^
Jeppesen (60)	ePatch	EKG	EMU	Focal, GTCS	43 (125)^f^	93.1^f^	1.1^f^
Vandenncasteele (61)	ByteFlies	EEG (behind ear)	EMU	Multiple	54 (182)	69.1	0.49^g^

*Studies before 2015 or reporting earlier results for a device in an identical setting were excluded. GTCS, generalized tonic clonic seizure; CP, complex partial; FT, fronto-temporal; TCS, tonic clonic seizure; FIA, focal impaired awareness*.

a *With optimal placement of device over the belly of the bicep*.

b *Best performing of three candidate algorithms*.

c *Three additional patients and 27 additional seizures reserved for training*.

d *Results reported are for the best performing of two algorithms considered using a patient-wise cross validation*.

e *Estimated from reported 93.7% specificity, assuming independent 5-min detection windows*.

f *Results reported are from the 53% of the cohort who exhibited adequate HR response to seizures*.

g *Results reported are from a patient-specific detection algorithm, which performed better than a cross-patient algorithm*.

Arm-band style wearable biosensors are available (Biofourmis, BrainSentinel), and many adhesive wearable sensors can be placed on the arm (Byteflies). This is a prime location for measuring EMG, and muscle activity can be used as a proxy for convulsive seizure activity. This placement may also facilitate simultaneous EKG measurement if wires are run through the sleeve to the subject's chest, although this may create maintenance challenges for long-term use. Arm band sensors can be rated lower in comfort and acceptability by subjects (62), and data quality may suffer due to movement or the sensor sliding slightly during wear. Small sensors affixed by adhesive patches (ByteFlies, EpiLog) can conceivably be placed anywhere on the body, although hair and perspiration may interfere with adhesives. Adhesive failure and skin irritation are a barrier to long-term (multiple weeks) and ultra long-term (months to years) use of these devices, although these devices may be suitable for prolonged (up to 7 days) monitoring (e.g., baseline seizure diaries). This category may be the most flexible sensor type, and there are commercially available research-quality sensors for EEG, EMG, EKG, PPG, accelerometry, and EDA (ByteFlies, Epilog). Continuous glucose monitor (CGM) and flash glucose devices, FDA and EU approved for diabetes monitoring, fall in this category of body-worn sensors and have reached a high level of technical maturity and reliability (63, 64).

In addition to mature, commercially available wearable biosensors, numerous early-stage sensors are under development, which may find application in epilepsy. Sweat sampling sensors are being developed for exercise applications and can non-invasively measure glucose, lactate, sodium, and other metabolites as well as drug or medication levels excreted in sweat. Fluidic sensors with similar technology have been integrated into mouth guards (65) and fabrics (66) to sample saliva and other bodily fluids. Google subsidiary Verily Inc. developed a contact lens with integrated glucose sensors, but abandoned the project in 2018 citing inconsistent monitoring results (67). Known hormonal and metabolic factors that may be altered prior to or immediately following seizures include melatonin (68), cortisol (69), reproductive hormones (70), prolactin and growth hormone (71), lactate, glucose (72), tRNA fragments (73), and others (74), thus providing a range of possible biomarkers for seizure detection. The field is evolving rapidly, and many innovative new sensors will likely become available. Hopefully, new sensor technologies will allow for the detection of a broader range of seizure types, beyond convulsive events.

### Behavioral Monitoring

Beyond sensing of basic biosignals, wearable devices and smartphones can be used to track behavior at more complex levels, including activity patterns, movement range, sleep duration and quality, and behavioral indicators of mood, for example, based on analyses of movement speed, social connectivity, or affective tone of speech (75–77). This opens up a window to analyzing behavioral changes occurring over days or weeks, which may correlate with seizure risk as suggested by studies on prodromes and on seizure precipitating factors (78, 79). Beyond passive monitoring, smartphones can be used to track mood changes and cognitive function by actively querying the user (80). Assessments can include pop-up questionnaires at predefined times, as well as specific test batteries assessing general cognitive capabilities like attention or working memory, thus capturing high level dynamic brain states. The use of a smartphones also allows for behavioral intervention, which is becoming a prominent adjunct therapy (81).

## Designing and Connecting Seizure Management Systems: Decreasing Barriers to Use

Despite a general willingness of people with epilepsy, caregivers, and healthcare professionals to use seizure monitoring devices (55), there are significant user requirements that impede long-term use. Johansson et al. concluded that on average 19% (range 6–24%) of data recorded from wearables in free-living environments may be missing due to a combination of technical and human factors (82). Cohen et al. found in a long-term study of wearables in Parkinson's and Huntington's disease that app-based reminders (“push notifications”) are useful tools in increasing continued device adherence (83), which could provide similar outcomes in seizure monitoring.

The aesthetics and comfort of devices are significant considerations in improving long-term adherence with wearable devices. Bruno et al. found that smartphone and watch-based devices were acceptable to over 70% of people with epilepsy; however, leg, upper-arm, chest, and head-based systems had <50% acceptance. Ring-style wearables had over 60% approval (84). Interestingly, there is a strong discrepancy between the views of people with epilepsy and caregivers for wristband and ring-style wearables, although why this is so is unclear (55). Performance characteristics are significant as well, and Patel et al. (85) showed a strong preference among people with epilepsy and caregivers for excellent sensitivity and text message alerts over comfort, battery life, and other features. Unfortunately, Patel et al. do not separate their responses between people with epilepsy and their caregivers, making it unclear if there are differences in view between these two groups. Furthermore, it is not clear that device users' reported preferences are truly predictive of their behavior. Janse et al. showed significant differences between the preferences of people with epilepsy and caregivers in device form factors, device accuracy, and seizure forecast range (10). Charging the batteries of wearable devices presents a considerable adherence challenge, as devices are ideally worn continuously through both sleep and wakefulness (55). It is estimated that 38–60% of users claim to be satisfied with recharging a device at least daily (55, 85). Battery charging depends on frequency of data sampling and telemetry. Many commercial fitness tracker devices upload a limited data stream to cloud platforms in near-real time using smartphones connected to the internet, either through broadband WiFi or mobile 4G. This approach is attractive for immediate feedback to people with epilepsy and caregivers but would pose a considerable burden on the battery of both the wearable device and smartphone for clinical quality sensor data on the order of 100s of samples per second.

### Computation and Connectivity

**Figure 2:**
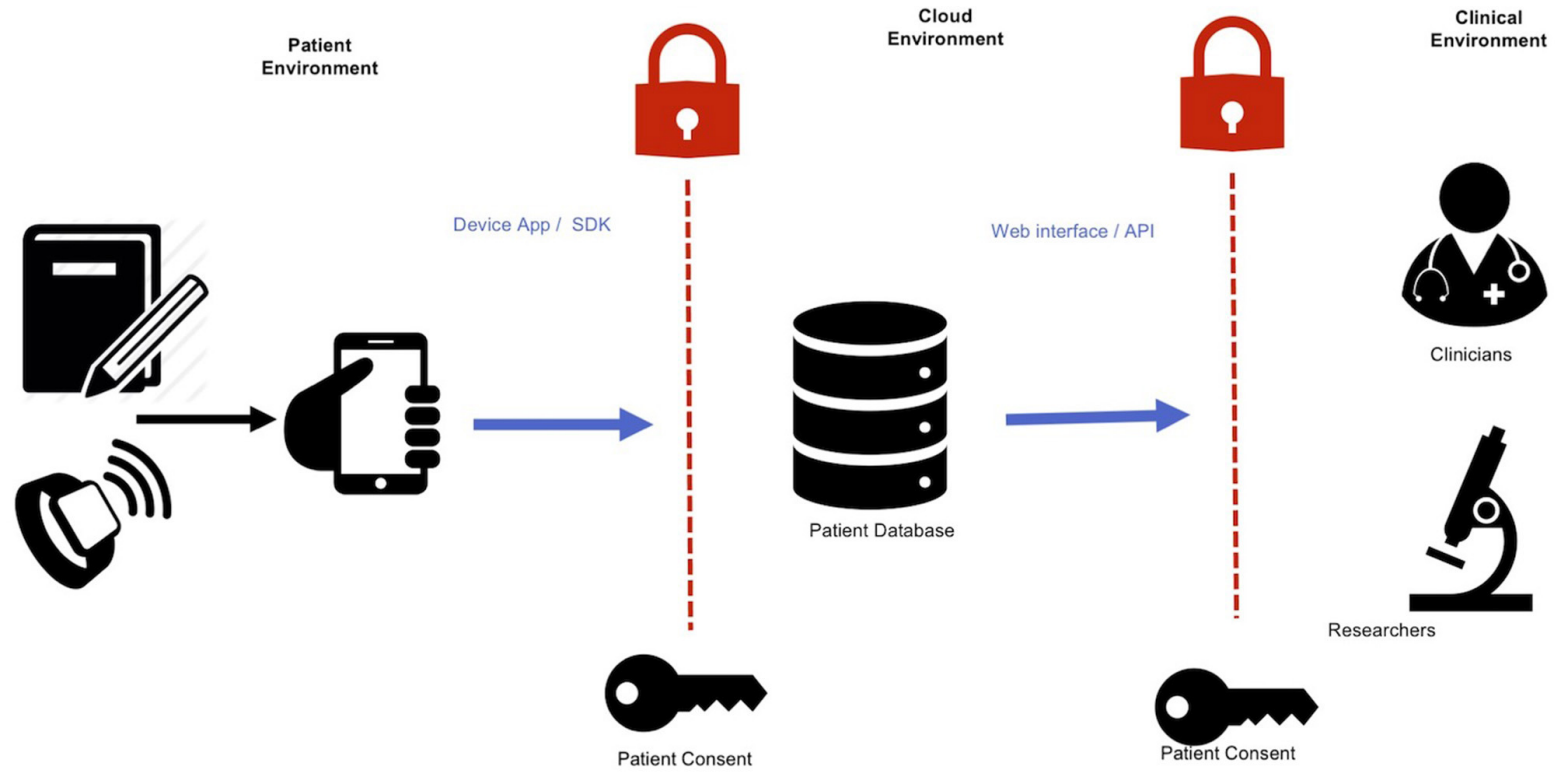
Integrating device data into online accessible databases. An abundance of data relating to physiology, behavior, and environment can be collected with wearable devices and smartwatches. These can then be collected into a single repository in cloud-based data storage. These data can then be accessed by relevant clinicians and researchers through a web interface or programmatic access. Informed permission is necessary for each step of data transfer: from user to the database, and from the database to the clinical environment.

Connecting data sources through cloud technologies has the potential to create insights into seizure patterns (86) and even forecast seizure events (9, 87). Measurements of a person's environment, physiology, and behavior through smartphones and wearables can be used to make patient-specific models that help clinicians understand individuals' risk factors (88). The following sections outline common methods for accessing data sources (such as seizure diaries or wearables) through cloud technologies. Figure 2 presents an overview of how various software interfaces may interact with device data.

Modern on-demand computing services make the collection and distribution of large datasets of potentially unknown size, expanding in response to the users' needs (14, 89). Importantly, data sources should use common formats and definitions for data storage, particularly for imperative entries such as timestamps, seizure type descriptions, and definitions of seizure durations (90). Without accurate timing information, errors can occur between data sources, creating noisy repositories, and inaccurate forecasts. Removing differences between data sources minimizes barriers to integrating heterogeneous data streams and gives the best opportunity for improved care.

Machine learning and artificial intelligence methods are integral to seizure detection and forecasting with wearable biosensors. Machine learning approaches allow algorithms to adaptively learn patterns in data, which may not be apparent to the human observer. Traditional machine learning requires preprocessing raw data to extract features or characteristics of interest, which are then normalized and passed to a classification algorithm for analysis. However, deep learning, or convolutional neural network approaches, provides “end to end” learning, where extraction of salient features is handled by the initial layers of the neural network after repeated presentation of training data (91). Automatic feature extraction is considered a key advantage of deep learning for seizure prediction, because it enables an algorithm to be tailored to particular seizure types or even an individual seizure semiology (or semiologies) (92, 93). Despite this ability for automated feature extraction, the signals recorded must contain some fundamental information relating to seizure events, and hence, appropriate device and sensor selection is still required for utility. For a discussion on factors that may contribute to seizure likelihood, see Section Factors contributing to seizure likelihood. A hurdle for machine learning, and deep learning in particular, is that algorithms typically require a very large number of training seizures in order to learn a generalized representation of the data. Epilepsy databases have facilitated development of machine learning and deep learning methods for seizure detection (94) and forecasting (95–99). This “big-data” approach may improve accuracy in detecting more challenging seizure types.

## Forecasting Seizure Likelihood

People with epilepsy consistently rate the apparent unpredictability of their seizures to be the most disabling aspect of their condition (10, 11), and a reliable system to forewarn individuals or caregivers of impending seizures could allow fast-acting medications to be administered, or simply allow a person to take preparatory measures. To date, most devices for use in epilepsy monitoring have been focused on seizure detection, where the main utility from the perspective of people with epilepsy is providing a seizure alert to their clinicians or caregivers. In this context, false alarms have the potential to be disruptive to the life of someone with epilepsy, their families, and caregivers, and can cause people to stop using seizure detection devices (100). However, in seizure forecasting applications, the primary end goal is to inform the individual of their current seizure likelihood. This context reduces the impact of “false alarms,” as not every high likelihood alert would be expected to result in a seizure (92). When evaluating forecasts, probabilistic measures can be used instead of only counting “hits” and “misses.” Therefore, although the problem of seizure forecasting is more complex than seizure detection from a signal analysis perspective (101), wearable devices may have broader application and wider acceptance in seizure forecasting, which will allow people with epilepsy to plan daily activities and take measures for seizure control. One retrospective validation study of seizure forecasting with wearables recently reported better than chance results in 30 of 69 (43.5%) in-hospital patients studied (102), confirming that forecasting with non-invasive devices is possible for many patients. The ability to record continuous, outpatient data from wearables will enable long-term tracking of risk factors and should improve forecasting performance.

Instead of trying to predict the exact time of an upcoming seizure, it may be more feasible to estimate the probability of someone having a seizure and communicate this risk in a clinically useful manner (12, 92, 103, 104). Accordingly, there is increasing interest within the clinical epilepsy community to develop seizure forecasting devices and applications (9) and understand user requirements (10, 105). In a survey-based study, Schulze-Bonhage et al. reported that probabilistic forecasts were generally considered equally useful to predicting exactly when a seizure would occur (105). They also found that missed seizures were considered worse than false alarms, and perfect accuracy was not considered a requirement for a forecasting device (105). This survey agrees with reports from individuals enrolled in the human study of a long-term seizure forecasting device (the NeuroVista trial) (2). Subjects in the NeuroVista trial reported on the usefulness of the device (106, 107), despite less than perfect sensitivity and time-in-warning of up to 30% (2). More recently, Janse et al. also showed that seizure forecasting devices were deemed broadly acceptable despite the potential for inaccuracy (up to “inaccurate 30% of the time”) (10). Externally worn devices were ranked more highly than subcutaneous or implantable devices (10), reinforcing the potential for development of wearable devices for seizure forecasting applications.

The development of qualitative and clinically useful metrics to evaluate seizure forecasts has been a key priority. Probabilistic measures can be used to evaluate performance (86, 108, 109), but defining an alarm threshold is often deemed necessary to determine clinical utility and the system becomes parameterized by the alarm duration, or “seizure prediction horizon” (110). Nevertheless, evaluation of false alarms is challenging because there is significant individual variability between seizure prediction horizons (2), and the “time-in-warning” is frequently reported as a proxy for a false alarm rate (2, 95, 111). In addition to benchmarking performance, it is important to understand user requirements for a forecasting interface (112). A recent study surveyed people with epilepsy and caregivers about the visual design of seizure forecasts, finding a range of preferences, although graphs that provided some temporal context (i.e., seizure risk plotted over the course of a day or month as opposed to a “gauge”) were rated more highly (113). Ultimately, *post-hoc* studies and surveys can only provide an indicative measure of the utility and benefits of a seizure forecasting device. In a prospective setting, some people initially with interest in forecasting devices may find false alarms to be debilitating, whereas others who were initially skeptical about the benefits may find a forecasting device very helpful (107).

### Patterns and Rhythms in Seizure Probability

It is now understood that most people with epilepsy exhibit circadian and slower, multiday temporal cycles that modulate their seizure likelihood [see (17) for a recent review]. Recent studies have demonstrated impressive seizure forecasting performance using multiday cycles measured from implantable EEG (114, 115), although prospective validation is needed. Cycles of seizure likelihood can also be measured from self-reported seizure times (116), and for a subset of people, cycles measured from seizure diaries are predictive of the likelihood of electrographic seizures and epileptic activity (116, 117). Machine learning can also be used with historic trends from self-reported seizure diaries, which may be useful to forecast future reported seizures (118, 119). Both cyclic and machine-learning approaches have been shown to accurately forecast seizures (or, more specifically, seizure diary events) in both focal and generalized epilepsies. Despite inaccuracy in individual seizure reporting, long-term patterns and cycles may still be accurately inferred for many individuals (116, 117). Due to the indications for use of who can have implanted EEG for long-term recording, the existence of cycles has largely been validated electrographically in individuals with focal epilepsies. Continuously recorded biomarkers remain important to truly characterize underlying epileptic rhythms, without the inherent limitations and biases of self-reported seizure diary records (120).

Wearable devices and subscalp EEG have the potential to improve seizure diaries by providing objective data and complementary information to help eliminate noise. Objective seizure measures may capture more seizures, enabling cyclic patterns to be detected earlier and characterized more accurately (38). On the other hand, seizure detection with wearable devices currently has a high error rate, and has only been established for convulsive or motor seizures (48–50, 121), although progress is ongoing for other seizure types (51, 52, 93, 122, 123). The distribution of errors with wearables is likely to be different to the error distribution of self-reported seizure diaries. There may also be gaps or poor quality data due to non-adherence or charging issues (124). Wearable seizure detectors may perform better at night, when there are fewer movement artifacts, and when individuals are less likely to self-report seizures (3). Wearable devices also do not suffer from diary fatigue, or other uniquely human biases. Therefore, wearable devices may have the potential to improve the accuracy and completeness of the historic record of individuals' seizure times when used along with a seizure diary. A more accurate seizure count, or a combined forecast from the two data streams (86), may provide a higher performing forecaster for users. A better record of seizure times enables personalized forecasting models to be trained and validated more rapidly and with greater reliability.

### Factors Contributing to Seizure Likelihood

Combining multiple sources of information, including cyclic patterns, EEG features, and other environmental factors, may contribute to a stronger forecast of seizure likelihood than any individual signal. There are many data sources that are readily available and have been shown to be associated with seizure likelihood. For example, sleep quality (68), weather (125, 126), mood (78), and stress (45, 46) may all make seizures more likely. These environmental factors can be combined with information from seizure times that capture individuals' daily, weekly, or monthly cycles to deliver an individualized forecast of seizure likelihood (12, 92, 104).

**Figure 3:**
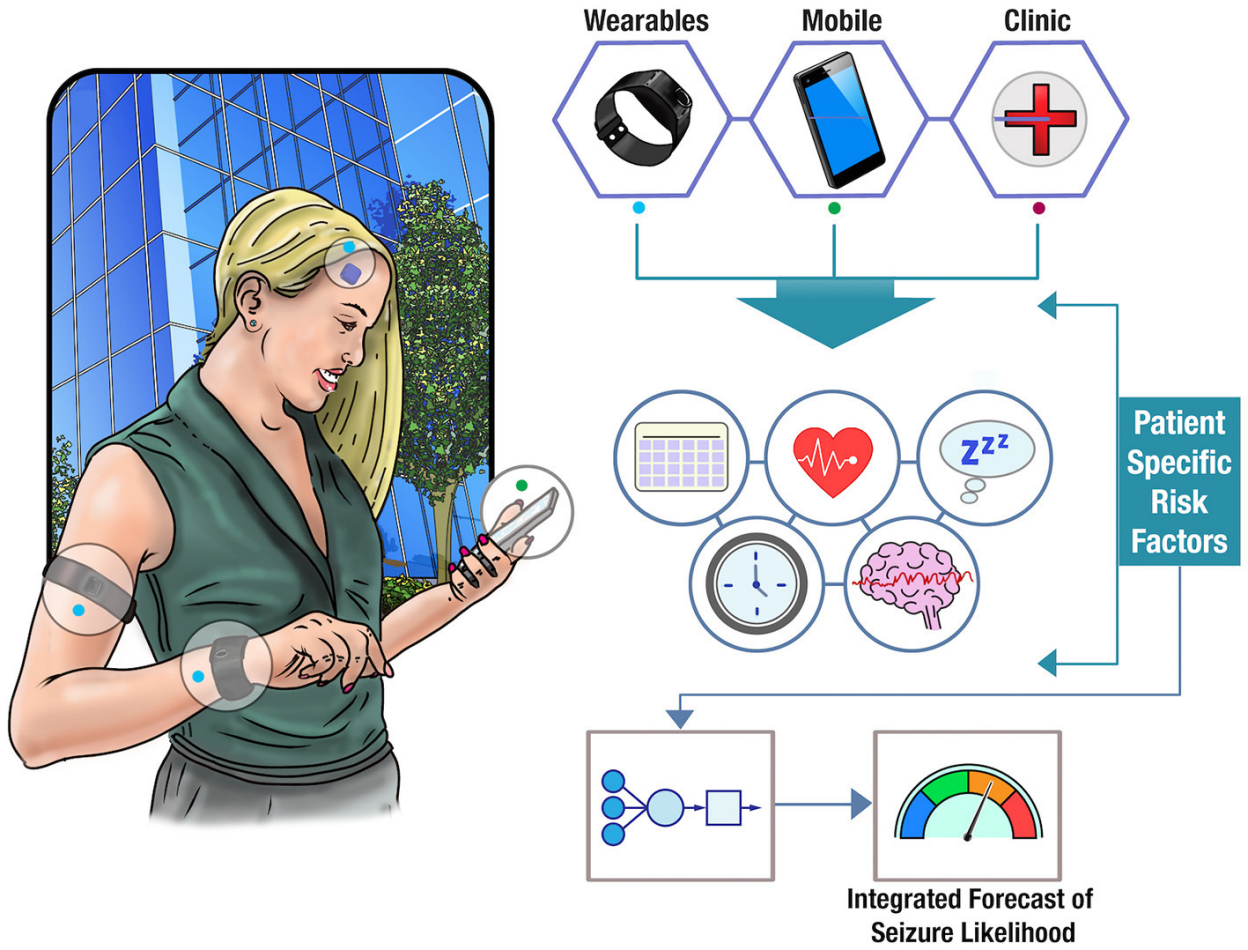
Forecasting seizure likelihood. The schematic shows how data from clinical notes, wearable devices, and mobile apps can be combined to obtain a deeper understanding of patient-specific risk factors. Utilizing cloud computing, these factors can be integrated into an individualized model of seizure likelihood and displayed as a real-time forecast to a user.

Wearable devices provide an opportunity to augment seizure forecasts with a growing number of physiological signals relevant to seizure likelihood. For instance, changes in heart rate have been often found to precede seizure onset by several minutes (127). Billeci et al. found that heart rate variability could be used to predict seizures up to 15 min before onset with >80% sensitivity, albeit with a relatively high average false positive rate of 0.41 per hour (almost 10 per day) (128). Signals that show some well-defined ictal changes, such as EDA (129–131), heart rate, or EMG (121), also show predictive changes prior to seizure onset (32). For instance, a recent study found predictive value in wearable sensor recordings EDA, blood volume pulse, accelerometry, and skin temperature (102). Average heart rate has also been found to show similar circadian and multiday cycles to epileptic activity, which are comodulated with seizure risk (132). Long-term datasets that record large numbers of individual seizures over long periods of time in conjunction with continuous wearable monitoring data promise to shed new light on these patterns governing autonomic nervous system and metabolic activity that are co-modulated with seizure onset. The potential of wearable monitoring to track individual seizure triggers may be more powerful when coupled with behavioral and mood data. Figure 3 illustrates the concept of a multi-modal seizure forecasting system.

**Figure 4:**
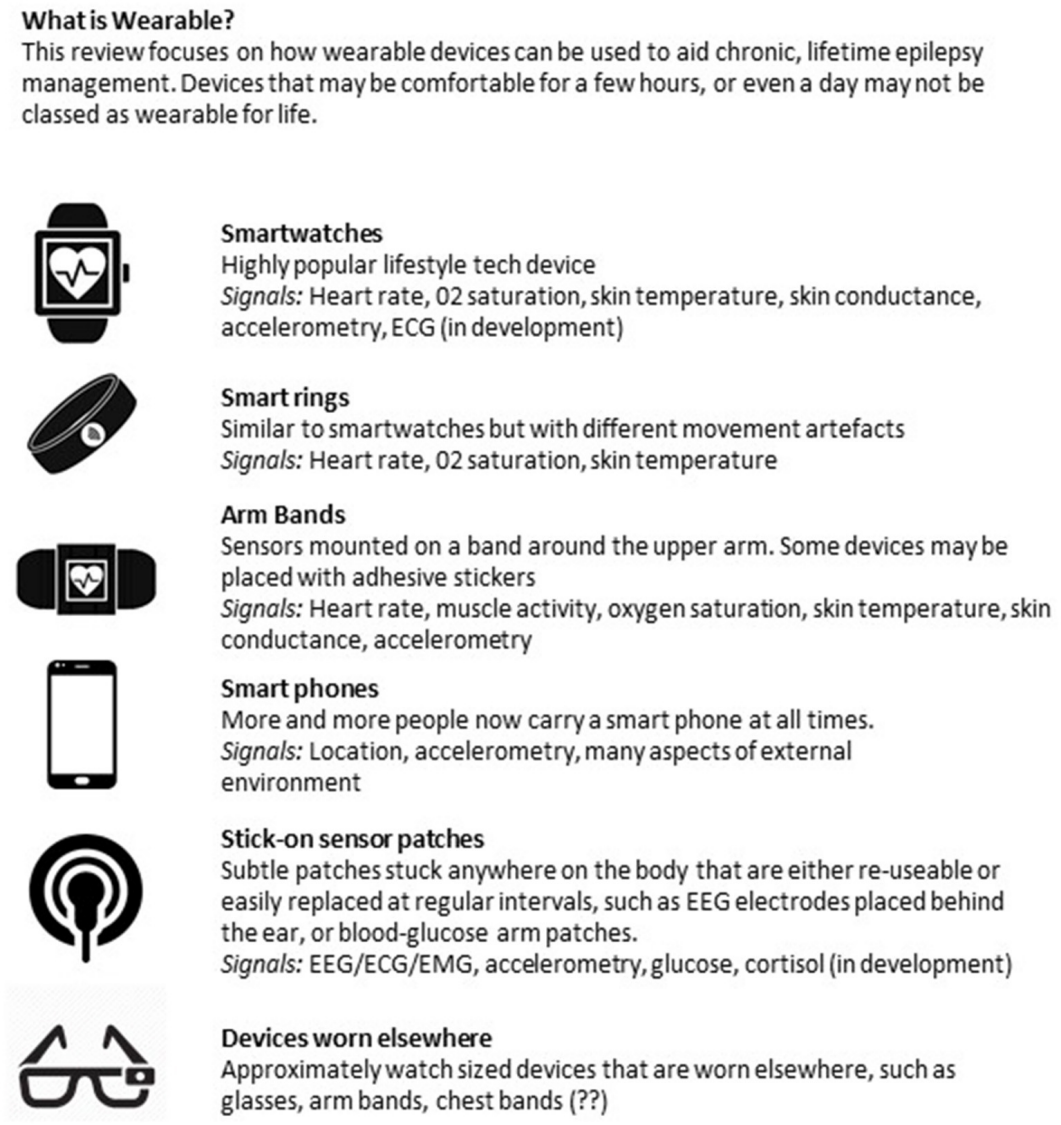
Overview of wearable devices in epilepsy.

There is some early promise that physiological signals derived from peripheral or autonomic systems (i.e., cardiac activity) contain relevant information for predicting seizure onset. Figure 4 illustrates a number of these systems. Currently, insufficient evidence exists that any stand-alone peripheral signal could be used as a seizure forecast with adequate sensitivity and specificity (105). However, with more data to determine patient-specific trends, and in combination with other predictive signals, wearable monitoring may contribute to an integrated forecast of seizure likelihood. As more prospective, clinical studies of forecasting systems are undertaken, a better understanding of the ideal signals, device specifications, user needs, and performance benchmarks will be elucidated, and forecasting systems may begin to reduce the burden of seizure unpredictability on people living with epilepsy.

## Conclusion

We are at the edge of a transition in the way that we identify, analyze, and manage seizures and epilepsy. At the moment, however, there is still relatively limited data on which to base decisions about the suitability of various devices currently available, and the types of seizures in which they might be best deployed, particularly for non-motor events. There are challenges ahead regarding the hardware, power, data, and security of the various devices available, though these problems are being solved in numerous mobile device applications. Significant challenges remain around issues related to usability of the systems, and certainly their chronic use. Developing concepts though relating to utilization of multiple modality streams and integrating this information will serve to provide accurate data on which to more effectively manage epilepsy in the clinic and evaluate new therapies. Ultimately, data from a variety of systems will contribute to seizure forecasts and enable people with epilepsy to achieve a greater degree of safety, freedom, and dignity.

## Author Contributions

BB, EN, and PK performed the literature search and literature revision, drafted the manuscript, reviewed, and edited for important intellectual content. MR, AS-B, GW, DF, MN, and SD participated in the interpretation of data in the literature, reviewed, and edited the manuscript for important intellectual content. MC developed the original concept and design of the manuscript, performed the literature search and analysis of data in the literature, drafted, reviewed, and edited the manuscript. All authors contributed to the article and approved the submitted version.

## Conflict of Interest

PK, EN, DF, and MC are employees of Seer Medical, which provides diagnostic EEG services. BB and GW have licensed IP to Cadence Neuroscience Inc., and have received research devices at no charge from Medtronic Inc. for a study. The remaining authors declare that the research was conducted in the absence of any commercial or financial relationships that could be construed as a potential conflict of interest.

## Appendix: Panels

### Panel 1: What Is Wearable?

This review focuses on how wearable devices can be used to aid chronic, lifetime epilepsy management. Devices that may be comfortable for a few hours, or even a day may not be classed as wearable for life. Furthermore, the term “wearable” implies a degree of accessibility that assumes no specialized medical knowledge is required for use.

### Smartwatches

Highly popular lifestyle tech device.

*Signals:* photoplethysmography, O^2^ saturation, skin temperature, skin conductance, accelerometry, location (GPS), EKG (in development).

### Smart Rings

Similar to smartwatches but with different movement artifacts.

*Signals:* photoplethysmography, O^2^ saturation, skin temperature.

### Arm Bands

Sensors mounted on a band around the upper arm. Some devices may be placed with adhesive stickers.

*Signals:* Heart rate, muscle activity, oxygen saturation, skin temperature, skin conductance, accelerometry.

### Stick-On Sensor Patches

Subtle patches stuck anywhere on the body that are either re-useable or easily replaced at regular intervals, such as EEG electrodes placed behind the ear, or blood-glucose arm patches.

*Signals:* EEG/EKG/EMG, accelerometry, glucose, cortisol (in development).

### Smart Phones

Smart phones are not strictly wearable but most wearable devices integrate and present information to users *via* smartphone. Furthermore, more and more people now carry their smart phone at all times, in a pocket or handbag.

*Signals:* Location, accelerometry, microphone, usage patterns, many aspects of external environment.

### Excluded Devices

This review does not consider most scalp EEG electrode caps or headbands to be wearable. Similarly, implantable devices may be eminently suitable for long-term use, but they are not considered “wearable.”

### Panel 2: Key Considerations in Wearable Device Design

- Comfort: devices should be able to be worn without discomfort for extended periods of time, including during sleep and activities such as exercise and bathing.
- Battery life: wearables should be able to record at least 24 h of activity without needing to recharge the device. Recharging time should be limited to a few hours. Connectors should be standard types such as micro-USB or USB-C.
- Accessibility: design of devices and associated phone apps should account for differences in age groups, genders, vision capabilities, and body sizes. Particular care should be given to testing PPG sensors on a variety of skin pigments.
- Appearance: devices should be inconspicuous, or otherwise not immediately identifiable as medical devices.
- Security: data security and individual anonymity are of high concern to users, caregivers, and clinicians. Different regions and jurisdictions will have unique requirements for security compliance, and laws regarding data access and ownership.
- Internet connectivity: Broadband or 4G internet is required to transmit data efficiently. Devices can only store a limited amount of data internally before requiring upload to an associated device (e.g., *via* Bluetooth) or the cloud.
- Integration: interfacing with other devices is essential. Connection with resources such as diaries, smartphone sensors, and location is becoming ubiquitous.

### Panel 3: Case Study: A Bump in the Dark

A 30-year-old man presented with a generalized tonic–clonic seizure after over 12 months of seizure freedom on 500 mg Sodium Valproate once daily. He complained of 6 month history of occasionally feeling poorly rested. An MRI appeared normal, and he was otherwise generally healthy. After two 7-day video-EEG studies (first non-diagnostic), a GTCS was captured lasting 2 min occurring during sleep, of which he had no memory. An additional 500 mg Sodium Valproate was given in the evening and began wearing a wrist-worn accelerometry device to detect nocturnal seizures in the home. Three events were detected within a 2-month period, none of which could be recalled. Carbamazepine was commenced, and he has since been free of seizures for over 6 months.

This case presents a common problem—a seizure diary that provides little information to guide treatment, and non-diagnostic, time-consuming video-EEG studies. Monitoring within the home can provide longitudinal seizure counts with reasonable sensitivity for GTCS events (from both wakefulness and sleep) and provide near real-time alerts to caregivers. Without a wearable device, multiple video-EEG studies may be required to measure the effect of each of the additional medications.
